# The Effects of Temporal Contiguity and Expertise on Acquisition of Tactical Movements

**DOI:** 10.3389/fpsyg.2020.00413

**Published:** 2020-03-13

**Authors:** Aïmen Khacharem, Khaled Trabelsi, Florian A. Engel, Billy Sperlich, Slava Kalyuga

**Affiliations:** ^1^LIRTES (EA 7313), UFR SESS-STAPS, Paris-East Créteil University, Créteil, France; ^2^DeVisu (EA 2445), Polytechnic University of Hauts-de-France, Valenciennes, France; ^3^UR15JS01, Education, Motricité, Sport et Santé (EM2S), High Institute of Sport and Physical Education, University of Sfax, Sfax, Tunisia; ^4^Institute of Sport and Sport Science, Department Movement and Training Science, Heidelberg University, Heidelberg, Germany; ^5^Integrative and Experimental Training Science, Institute for Sport Sciences, University of Würzburg, Würzburg, Germany; ^6^School of Education, University of New South Wales, Sydney, NSW, Australia

**Keywords:** multimedia learning, temporal contiguity, expertise, video-audio sequence, basketball, novices and experts

## Abstract

Various studies demonstrated that multimedia learning improves when text and pictures are presented contiguously in time rather than separately – the temporal contiguity effect. The present study investigated whether this advantage is restricted to only novice learners (novices) or also extends to more knowledgeable learners (expert), and whether it depends on the length of instructional segments. Learners with varied levels of expertise (experts vs. novices) learned about basketball game system in five different experimental conditions. In the first three conditions, an entire video clip and audio text were presented either at the same time or the video clip was presented before or after the entire audio (macro-step presentations). In the remaining two conditions, short segments of the video clip were presented before or after corresponding short segments of the audio (micro-step presentations). Overall, novice learners benefited more from the concurrent presentation (combination of learning and mental effort scores); in addition, and in the case of macro-step presentations novices performed better when the audio segment preceded the video clip segment. However, experts benefited more from the micro-step presentations, demonstrating an expertise reversal effect.

## Introduction

Recent advances in information technology and graphics have enabled the production of powerful and effective learning settings. With these developments, we can easily present words in the form of narrated discourse or on-screen text, and we can present pictures in the form of still photographs, animations or video clips. However, simply presenting information by using multiple modalities does not guarantee performance, particularly when limitations of the human cognitive architecture are not taken into consideration (Sweller et al., 1998, 2019). For instance, there is a large body of evidence suggesting that effectiveness of multimedia instruction increases when words and pictures are synchronized in time, that is, presented simultaneously rather than successively. This well-established finding is known as the “*temporal-contiguity effect*” (Mayer and Anderson, 1992; Ginns, 2005; Mayer, 2009).

The cognitive theory of multimedia learning (CTML, Mayer, 2005) provides a theoretical framework for explaining the temporal-contiguity effect. CTML is a theoretical framework that allows deriving design principles for multimedia learning. It is based on the idea that there are two separate systems for processing auditory/verbal and visual/pictorial information, both limited in their processing capacities and duration. Understanding an illustrated document requires the selection and organization of the relevant elements (pictures and/or words), the activation of relevant prior knowledge, and the construction of links between the individual’s verbal and pictorial mental models of the information beforehand, so that the two models can be integrated with each other and also with prior knowledge. This integration is expected to be easier if the two sources of information that should be linked together are held in working memory at the same time (temporal contiguity, see Ginns, 2006; Mayer, 2009, for an overview). For example, in a study of Mayer and Anderson (1991), participants were asked to view an animation that showed how a tire pump works. The narration was presented either simultaneously with the animation or sequentially (i.e., either before or after the animation). Results showed that learners in the simultaneous group obtained better learning results than learners in the sequential groups. The temporal contiguity effect has often been replicated with different materials and procedures, resulting in the general recommendation to avoid the non-contiguous presentation of words and pictures in time (for similar results, see also Mayer and Anderson, 1992; Mayer and Sims, 1994).

However, in a more recent study, Schüler et al. (2012) asked students to view pictures on the development of tornados, which were accompanied by either spoken or written explanations presented either simultaneously with, before, or after the pictures. The results demonstrated that simultaneous spoken presentations were not superior to sequential spoken presentations, which contradicted the temporal contiguity assumption. An explanation for this lack of a temporal contiguity effect for spoken text might be the high attentional requirements related to the learning task. According to CTML, if visual search associated with identifying the pertinent information in the diagram proves to be particularly complex, the learners will be unable to build effective connection between the spoken explanation and the corresponding visual (Jamet et al., 2008). Consequently, fewer processing resources will be left for learning, thus canceling the temporal contiguity effect.

Another potential moderator for the temporal contiguity effect is the length of instructional segments. Mayer et al. (1999) asked participants to view an animation depicting either the process of lightning formation or how car brakes work, which was accompanied by an entire narration presented simultaneously with, before, or after the entire animation (successive large bites), or short portions of the narration were presented before or after the corresponding short portions of the animation (successive small bites). Overall, the results showed that simultaneous animation-narration presentation can lead to better learning outcomes than sequential animation-narration presentation only if the animation-narration segments are not too short in length.

A closer look at the aforementioned studies reveals that the temporal contiguity principle has only been investigated using students with low levels of prior knowledge (novices) as participants, and it is yet unknown if this principle applies to learning by students with higher levels of prior knowledge (experts). The phenomenon, in which the effect of an instructional principle differs for learners with different levels of prior knowledge, is referred to as the expertise reversal effect (Kalyuga et al., 2003; Kalyuga, 2007; Khacharem et al., 2013, 2015). In accordance with general cognitive studies of expert-novice differences (e.g., Chase and Simon, 1973; Starkes and Ericsson, 2003), studies on the expertise reversal effect have found that many instructional design techniques that are highly effective with less knowledgeable learners, lose their effectiveness and can even have negative consequences when used with more experienced learners, and vice versa.

Based on the above analysis, the current study was designed to investigate the influence of levels of learner expertise and the length of learning segments on occurrence of the contiguity effect in multimedia learning. The study was conducted in the sport and exercise domain using learning materials in team basketball. Basketball is a ball game of two teams that compete against each other. Each team consists of five field players. The winner is the team with more baskets in 40 min (four periods of 10 min). The use of a basketball activity as leaning setting is attractive given its dynamic nature and the complex existing interactions between elements of play (Khacharem et al., 2019a). In this study, tactical instructions for a game episode were provided in several different formats: the entire video clip and audio of the episode were presented at the same time; the entire video clip was presented before or after the entire audio (macro-step sequential presentations); for each short event from the game episode, a video segment of the event was presented before or after the corresponding audio segment of the event (micro-step sequential presentations).

According to CTML, temporal contiguity of information can enhance deep learning when corresponding visual and verbal representations are held in working memory at the same time. Thus, we hypothesize that the concurrent presentation would lead to more efficient learning than the micro-step sequential presentations, which in turn would lead to more efficient learning than the macro-step sequential presentations (Hypothesis 1). The rationale for this prediction is that if more connections between the corresponding parts of audio and video presentations were close in time, then more learning would be fostered.

Furthermore, if presenting audio before video clip required the same cognitive resources to that found when presenting audio after video clip, we might expect that these two formats of presentations would show the same pattern of results (Hypothesis 2a). Alternatively, if significant effort is required to create effective links between verbal and spatial information, presenting audio before video clip should yield better performance (i.e., combination of learning and mental effort scores) than presenting video clip before audio (Hypothesis 2b). This alternative prediction is consistent with the verbal facilitation effect (e.g., Huff and Schwan, 2012) based on the assumption that spoken instructions (audio) could effectively guide visual attention to the relevant parts in the visual scene (video clip), which may enhance learning processes. Thus, in terms of the order of sequential presentations of corresponding audio and visual segments, these two alternative hypotheses could be tested in the present study.

Finally, in relation of levels of expertise, because of previously acquired schemas, we might predict that expert learners would perform at the same level and invest the same amount of mental effort regardless of the type and order of presentation (Hypothesis 3).

## Materials and Methods

### Participants

Forty-four team-basketball players with different levels of experience were recruited to participate in this experiment. Four participants were consequently excluded from the analysis, due to incomplete participation of experimental sessions. Finally, 20 novices (age: 26.00 ± 2.88 years; *SD* =), and 20 experts (age: 26.6 ± 2.34 years) were included in the study. The novices were students from the local university who were familiar with the rules of the game, but had never played a team sport in a club. The experts were professional players engaged with teams from the third division of the French basketball league. Expert participants had been playing basketball competitively for 12.45 ± 2.45 years. After receiving a full description of the protocol, including potential risks and benefits, participants gave their written consent to participate in the study. The present study was conducted according to the Code of Ethics for human experimentation, the Declaration of Helsinki (World Medical Association, 2013) and the protocol was fully approved by the ethics committee of Paris-Est University before the commencement of the assessments. All participants reported normal or corrected-to normal levels of visual function.

### Experimental Materials

The experimental films consisted of five attack systems developed in collaboration with two experienced basketball coaches (mean age: 47 years, mean experience as basketball coach: 13.25 years). The sequences were filmed from a camera position 3 m (Canon XM-2, made in Japan) above the ground from the middle of the field such that all players were visible. The camera did not zoom or pan during recording. All video clips showed the evolution of a basketball system. Each video clip comprised four phases of play and related spoken texts explaining the movements of five attacking players #1, #2, #3, #4, and #5) who carried out a coherent combination of play toward the opponent’s court before a basket was scored. Audio texts were presented by a male voice without foreign accent through loudspeakers. Subsequently, to guarantee that the developed materials provided a realistic depiction of a basketball game, for each sequence, another two experienced basketball coaches independently rated on a 5-point Likert scale the degree of representativeness of each attack system (0 = very non-representative; 5 = very representative). All sequences were rated 4 or above and thus were considered suitable for the experimentation.

For each video clip, five versions for different experimental conditions were generated. The concurrent presentation (C) consisted of a video clip depicting the attack system along with simultaneous audio text describing each step. The related non-concurrent presentations included four versions: VA presentation consisted of the entire video clip followed by the entire audio, whereas the AV presentation consisted of the entire audio followed by the entire video clip. These two presentations refer to macro-step presentations. The VA + presentation consisted of a series of short video clip segments (depicting sequential phases of the play) each followed by the corresponding audio segment (depicting the same phases of the play), whereas the AV + presentation consisted of a series of short audio segments each followed by the corresponding video clip segment. These presentations refer to micro-step presentations.

### Procedure

Each participant visited the experiment place (i.e., a basketball pitch in a gym) a total of five times. The participants were randomly allocated to experimental conditions using a counterbalancing system to minimize order effects. Each experimental session commenced approximately at the same time of day (to minimize any circadian variations) and lasted approximately 20 min on each occasion. A minimum of 48 h of interval between sessions (visits) was given to each participant. At the beginning of the first visit, all participants received written information about the experimental procedures and signed a consent form. Then, they answered demographic questions and the domain-specific prior knowledge test. Afterward, depending on the experimental condition, one of five versions of experimental film was projected on the screen and participants were instructed as follows: “*Dear Participant! In the following, a situation representing a basketball game system will be presented. The situation will be presented only once and for a limited time. Please try your best to memorize it as well as possible. Press the space bar to start the presentation!”* After participants pressed the space bar, the learning phase started. When the projection was over, participants were asked to perform experimental tests, which measured first mental effort, then verbal recall, and finally pictorial recall of the respective experimental film.

*Invested mental effort* in studying each learning material was assessed with a 9-point rating scale (Paas, 1992) ranging from 1 (very, very low effort) to 9 (very, very high effort) with participants being asked to indicate how much mental effort did they invest in studying the instructional material. *Verbal recall* was assessed with nine multiple-choice questions (e.g., “After player #3 stops dribbling, he passes the ball to player #?”) and three open questions (“What is the purpose of the screen made by player #2”). Multiple-choice items required the selection of the correct answer out of four provided possibilities. The open questions required the recall of one single fact mentioned in the learning material. Two independent raters coded the answers to the open questions. The inter-rater reliability analysis showed almost perfect agreement, with a Cohen’s κ = 0.92; disagreements between raters were resolved by consensus. *Pictorial recall* was assessed by asking participants to reconstruct the game system on a recall response template that had the same computerized visual perspective as the scene presented in the corresponding video clip. Specifically, participants were provided with an empty basketball half-field and instructed to reconstruct as accurately as possible the corresponding phase of play (i.e., phase 1). They used a computer mouse to drag and drop the symbols from outside the represented field to positions on the field. The number of symbols to be dragged by the computer mouse matched exactly the number of actions and players on the video clips. Once finished with the first phase, the participants had the choice either to continue the reconstruction task by clicking on “*Next”* bottom (if they believed that situation has not yet been completed), – in this case a second empty basketball half-field would appear, – or to click on the “*End*” button (if they believed that situation has been completed). An independent rater scored the total number of correct and incorrect steps that each participant included in his description of the system. Each correct response yielded 1 point; otherwise the participant received 0 point.

### Data Analysis

A mixed-design ANOVA was used to analyze the data. The between-participants factor was expertise (expert or novice) and the within-participants factor was condition (C, AV +, VA +, AV, or VA). For all analyses throughout this study, we used *p* < 0.05 as the criterion for significance, partial eta squared (η_p_^2^) values are provided as a measure of effect size for all main effects and interactions, partial eta squared values of 0.01, 0.06, and 0.14 represent small, moderate, and large effect sizes, respectively (Clark-Carter, 1997).

## Results

Descriptive statistics for verbal recall, pictorial recall, and mental effort for the experts and novices under each experimental condition are presented in Table 1.

**TABLE 1:** Means and (Standard Deviations) for verbal recall.

	Experts					Novices				
	C	AV +	VA +	AV	VA	C	AV +	VA +	AV	VA
Pictorial recall	75.13 (7.49)	83.31 (5.64)	81.62 (3.31)	67.26 (6.11)	64.15 (7.60)	58.81 (7.23)	52.55 (6.78)	47.87 (6.09)	35.01 (7.20)	28.51 (6.83)
Verbal recall	76.13 (7.49)	79.43 (6.06)	76.64 (4.76)	67.92 (6.11)	65.41 (7.60)	58.31 (7.23)	53.3 (6.78)	49.5 (6.75)	35.35 (7.20)	28 (7.88)
Mental effort	3.64 (0.70)	3.05 (0.89)	3.29 (0.91)	4.58 (0.87)	5 (1.17)	5.55 (0.88)	5.3 (1.03)	6.35 (1.18)	6.55 (0.99)	6.9 (1.11)

*Pictorial recall and mental effort for the experts and novices under each experimental condition.*

### Pictorial Recall Test

**FIGURE 1:**
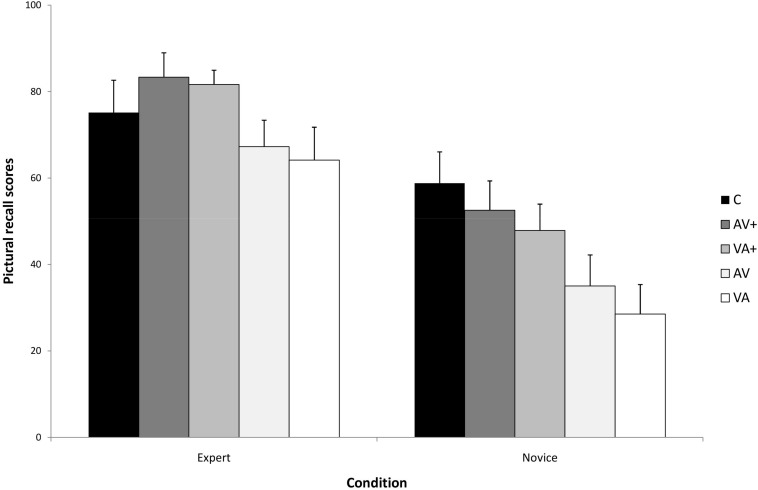
The Expertise × Condition interaction for recall pictorial recall accuracy.

The primary dependent measure of interest is performance on the pictorial recall test. Figure 1 presents the means and standard deviations by presentation conditions and levels of expertise on the pictorial recall test. The analysis indicated a significant main effect of presentation, *F*(4,140) = 35,34, *p* < 0.001, η_p_^2^ = 0.71, as well as a significant main effect of expertise, *F*(1,35) = 148,68, *p* < 0.001, η_p_^2^ = 0.85. The analysis also indicated a significant presentation by expertise interaction, *F*(4,140) = 13.28, *p* < 0.001, η_p_^2^ = 0.27, indicating that the differences in recall among the presentations vary as a function of expertise. A simple main effects test indicated a significant difference between presentations for novices, *F*(4,76) = 66,94, *p* < 0.001, η_p_^2^ = 0.77. A *post hoc* Bonferroni test showed that novices performed significantly better following the micro-step presentations than following the macro-step presentations (*p* < 0.001). Concerning the presentation order, the analysis showed that novices performed better only following the AV + presentation comparing to the VA + presentation (*p* = 0.06). Finally, novices performed significantly better following the concurrent presentation than in all the other presentations (*p* < 0.001). A simple main effects test indicated a significant difference between presentations for experts, *F*(4,64) = 35,78, *p* < 0.001, η_p_^2^ = 0.69. A *post hoc* Bonferroni test (α = 0.05) showed that experts performed significantly better following the micro-step presentations than in the macro-step presentations (*p* < 0.001), which did not differ significantly from each other based on the order of audio and visual components. Moreover, they performed better in the micro-step presentations than in the concurrent presentation (*p* < 0.001), indicating, when compared to the pattern of novice performance on this test, an expertise reversal effect.

### Verbal Recall Test

**FIGURE 2:**
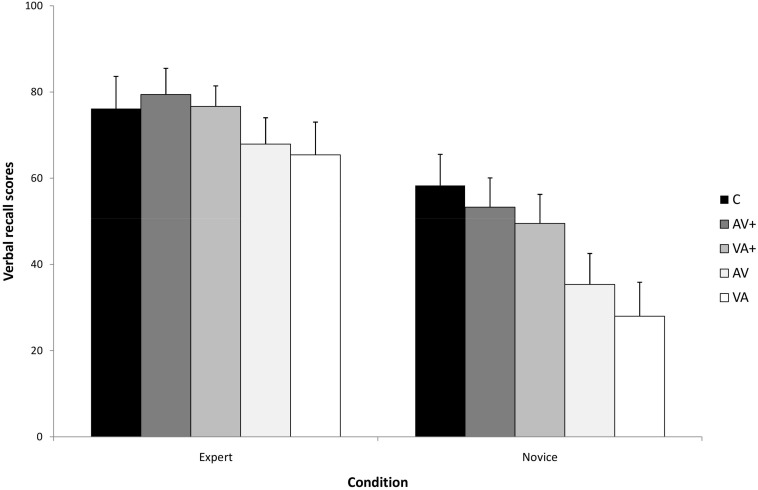
The Expertise × Condition interaction for recall verbal recall accuracy.

Figure 2 presents the means and standard deviations by presentation conditions and expertise levels on the verbal recall test. The analysis indicated a significant main effect of presentation, *F*(4,140) = 65,89, *p* < 0.001, η_p_^2^ = 0.64, as well as a significant main effect of expertise, *F*(1,35) = 48,68, *p* < 0.001, η_p_^2^ = 0.84. The analysis also indicated a significant presentation by expertise interaction, *F*(4,140) = 4.28, *p* = 0.008, η_p_^2^ = 0.21, indicating that the differences in recall among the presentations vary as a function of expertise. A simple main effects test indicated a significant difference between presentations for novices, *F*(4,76) = 56,33, *p* < 0.001, η_p_^2^ = 0.74. A *post hoc* Bonferroni test showed that novices performed significantly better following the micro-step presentations than following the macro-step presentations (*p* < 0.001). Concerning the presentation order, the analysis showed that novices performed better following the AV + presentation than the VA + presentation (*p* = 0.02). Finally, they performed significantly better following the concurrent presentation than in the all other presentations (*p* < 0.001) – except than the AV + condition (*p* = 0.19). A simple main effects test indicated a significant difference between presentations for experts, *F*(4,64) = 17,34, *p* < 0.001, η_p_^2^ = 0.58. A *post hoc* Bonferroni test showed that experts performed significantly better following the micro-step presentations than following the macro-step presentations (*p* < 0.001), which did not differ significantly from each other based on the order of audio and visual components. However, they did not show significant differences between the micro-step presentations and concurrent presentation (*p* > 0.05), indicating, when compared to the pattern of novice performance on this test, a partial expertise reversal effect.

### Mental Effort

**FIGURE 3:**
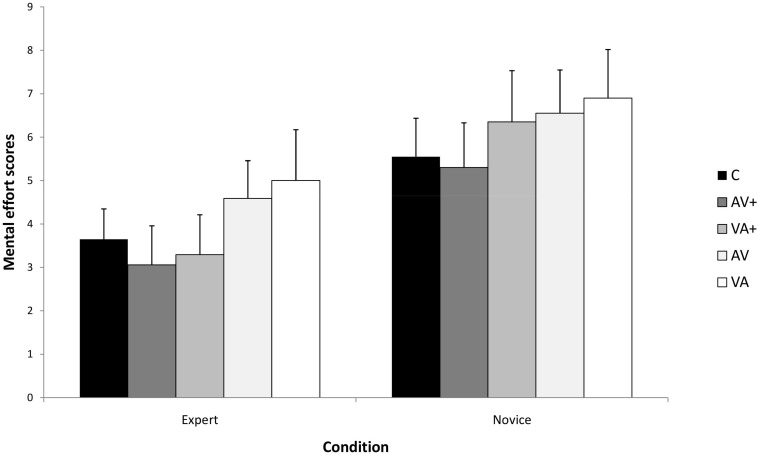
The Expertise × Condition interaction for mental effort.

Figure 3 presents the means and standard deviations by presentation conditions and expertise levels on the mental effort test. The analysis indicated a significant main effect of presentation condition, *F*(4,140) = 18,88, *p* < 0.001, η_p_^2^ = 0.35; and a significant main effect of expertise, *F*(1,35) = 135,68, *p* < 0.001, η_p_^2^ = 0.81. However, there was no significant presentation by expertise interaction, *F*(4,140) = 1.18, *p* = 0.09, η_p_^2^ = 0.03.

## Discussion

The experiment reported in this paper was designed to investigate the relation between levels of player expertise, the length of learning segments (macro- or micro-step presentations), and the effectiveness of temporal contiguity principle in the domain of basketball measured by the three dependent measures – visual recall, verbal recall and mental effort. As expected (Hypothesis 1), the results showed a temporal contiguity effect for novices, indicating that concurrent presentation was more effective (combination of learning and mental effort scores) than both micro- and macro-step presentations. According to CTML, the mental integration of spoken and visual information is facilitated when words and pictures are held in working memory at the same time. This should enable simultaneous processing of both information sources as well as their integration in working memory (see Ginns, 2006; Mayer, 2009, for an overview). Moreover, because the instructional messages delivered either through video clips or spoken words are ephemeral (i.e., transitory), important information necessary for successful recall performance may be missed when addressing only the visual or the verbal input system sequentially. Thus, concurrent presentations may reduce the harmful effects of information transience, as spoken words (audio) would act as verbal cues that guide learners’ attention to essential information, thereby reducing cognitive load involved in processing the learning material. Overall, the reported results confirm the robustness of the temporal contiguity phenomenon that was found in previous studies (Mayer and Anderson, 1992; Ginns, 2005), using a different sample of participants (i.e., players), a novel learning context (i.e., sport-related field), and realistic (video clips) rather than abstract representations of learning materials.

Moreover, the performance following the micro-step presentation condition was significantly better than that of the macro-step presentation condition; however, unlike the results reported by Mayer et al. (1999), it was significantly worse than the performance following the concurrent presentation group. It is possible that our experimental material was relatively more complex (the size of the segments exceeded the available working memory resources), and thus novice learners were not able to make as effective connections between spoken words and the corresponding segments of video clips, as when the video clips and audio were presented at the same time. For novices, the results also showed facilitating effects (but only in the case of macro-step presentations) when an audio commentary was presented before the corresponding video clip (Hypothesis 2b). It can be concluded that these participants could successively relate both verbal and visual information. That is, after having listened to verbal descriptions of a particular action of play (spoken words), novices were able to effectively identify (recognize) this information when it was depicted in the video clip, which might have facilitated its integration and subsequent transfer to long-term memory. These findings are in line with our Hypothesis 2b and confirm recent eye tracking studies in multimedia learning that suggested the text as the primary information source (as participants always started by reading text), while the pictures or video clips as a helpful addition (Schmidt-Weigand et al., 2010).

On the other hand, the results demonstrated that contrarily to novices, expert learners primarily benefited from micro-step presentations, thus indicating an expertise reversal effect – which partially confirmed Hypothesis 3. It is conceivable that these learners who had already acquired a relevant knowledge base due to extensive practice (Williams et al., 2011; Khacharem, 2017; Khacharem et al., 2019b), were able to develop, from the first pictorial or verbal segment, an effective mental representation of the corresponding phase of play. In this case, the information presented in the next segment might provide visual or verbal feedback which could be used by these learners to verify their mental representation. Even though these activities might generate additional intrinsic cognitive load, this is a productive load that would facilitate learners’ understanding of the sequence of play (and these expert learners should have sufficient working memory capacity to handle this additional load). However, in macro-step presentation conditions, experts failed to make effective referential connections between corresponding verbal (audio) and visual (video clip) presentations, since they were unable to hold the entire audio description until the video clip is presented (or to hold the entire video clip until the audio description is presented).

This study has a number of obvious limitations. Firstly, presenting the learning material only one time might have been not sufficient for effective learning, as evidenced by the fact that many participants – even experts – were not always able to successfully recall the game situation. It is therefore desirable to replicate the present results in learning environments that engage learners for more extended periods of time by allowing them to revisit learning segments. Secondly, the results are based on off-line measures (that is, memory-based performance measures and self-reports collected after the learning phase), which is common in research examining the acquisition of motor skills. In addition to these off-line measures, future research should also consider online measures such as think-aloud reports and eye-movement recordings to examine the underlying cognitive strategies. Thirdly, to our knowledge, this is the first study that investigated the relationship between temporal contiguity principle and levels of learner expertise. Replication studies using other domain areas, learning materials (e.g., with static pictures), and types of participants (e.g., gender) are necessary to further strengthen reliability to the reported results.

The findings of the present article suggest caution in the use of various types of multimedia presentations explaining the evolution of sport games (such as a basketball system) to players with different levels of expertise. Coaches and educators have long assumed that different training methods are required at different stages of performance to address the altering perceptual-cognitive mechanisms of the player (e.g., Beilock et al., 2002). The findings of the reported study begin to lend empirical support to this idea. According to the results, it is essential to apply the temporal contiguity principle (i.e., synchronize the presentations of spoken text and video clips) at the initial stage of learning in order to optimize learners’ comprehension of game situations. If this principle is ignored, novice players may experience heavy cognitive load resulting in inefficient tactical learning. However, at later stages of learning, i.e., with the increases in the amount of constructed and automated schemas, the temporal contiguity principle may turn into unhelpful strategy that impedes learning processes.

## Data Availability Statement

The datasets generated for this study are available on request to the corresponding author.

## Ethics Statement

The studies involving human participants were reviewed and approved by Research Ethics Committee of the Paris-Est University, France. The patients/participants provided their written informed consent to participate in this study.

## Author Contributions

All authors listed have made a substantial, direct and intellectual contribution to the work, and approved it for publication.

## Conflict of Interest

The authors declare that the research was conducted in the absence of any commercial or financial relationships that could be construed as a potential conflict of interest.
